# Strengthening school health in Namibia: A framework for policy development, implementation and evaluation

**DOI:** 10.4102/jphia.v17i1.1584

**Published:** 2026-06-09

**Authors:** Ndasilohenda Katangolo-Nakashwa, Faniswa Honest Mfidi, Kabwebwe Honoré Mitonga

**Affiliations:** 1 Department of Public Health, Faculty of Health Science and Veterinary Medicine, University of Namibia, Oshakati, Namibia; 2 Department of Health Studies, College of Human Sciences, University of South Africa, Pretoria, South Africa

**Keywords:** school health policy, student well-being, Namibia, equitable resource allocation, monitoring and evaluation, capacity building

## Abstract

**Background:**

Schools in rural Namibia face severe health challenges (malnutrition, infectious diseases, poor sanitation), leading to high learner absenteeism and poor academic performance. Current school health policies are often fragmented and insufficient to address these issues comprehensively.

**Aim:**

This study aimed to develop an evidence-based, contextually relevant conceptual framework to enhance school health policies in Namibia and similar resource-limited Southern African settings.

**Setting:**

The problem context is rural areas across Southern Africa. The resulting framework is specifically tailored for implementation in Namibia.

**Methods:**

This study employed a convergent mixed-methods design. A systematic review (following Preferred Reporting Items for Systematic Reviews and Meta-Analyses 2020 guidelines) synthesised evidence on African school health policy. Qualitative data were collected through 15 key informant interviews and 3 focus groups (*n* = 30) with key stakeholders, including educators, healthcare professionals and community members, using purposive sampling and thematic analysis.

**Results:**

The study developed a conceptual framework for Namibia. It integrates key components: evidence-based policy development, equitable resource allocation, capacity building and robust monitoring and evaluation. The framework emphasises stakeholder collaboration and addresses specific rural challenges, such as infrastructure gaps and inconsistent implementation.

**Conclusion:**

The proposed framework provides an evidence-based, contextually relevant approach to significantly improve school health policies in Namibia. It directly addresses the unique challenges of resource-limited Southern African settings.

**Contribution:**

The framework fills a crucial policy gap and offers a differentiated model focused on improving learners’ health and educational outcomes, moving beyond generic global approaches.

## Introduction

School health policies are critical for promoting the well-being and academic success of learners, particularly in low- and middle-income countries (LMICs), where health challenges are prevalent.^1,2,3,4^ In Africa, malnutrition, infectious diseases and inadequate access to healthcare and sanitation remain major barriers to learning.^5,6,7^ For instance, recent reviews indicate that malnutrition among school-aged children in LMICs ranges from 10% to 30%, with undernutrition strongly linked to poor cognitive development and absenteeism.^1,6,8,9^ Similarly, human immunodeficiency virus (HIV) and tuberculosis (TB) continue to exert a heavy toll on education systems, contributing to teacher and learner absenteeism and reduced school performance.^1^

Namibia exemplifies these challenges. According to the Global School-based Student Health Survey (2024), 13.6% of Namibian students aged 13–17 years are underweight, while 14.1% are overweight, and 4.1% are obese.^3,8^ Furthermore, Namibia ranks among the top 30 high-burden TB countries globally, and HIV incidence remains significant, particularly among adolescents (4.39 per 1000).^9,10,11^ These health burdens contribute to absenteeism and poor academic performance, especially in rural schools where infrastructure and health services are limited.^3,8^

Despite some progress, Namibia lacks a comprehensive, evidence-based school health policy framework that addresses these multifaceted challenges. Existing policies are often fragmented and reactive.^1,3,12^ To close this gap, this study aims to develop a conceptual framework for school health policy in Namibia, grounded in evidence-based policy development, equitable resource allocation and robust monitoring and evaluation (M&E). The proposed framework emphasises stakeholder engagement, including educators, health professionals and communities to ensure contextual relevance and sustainability.

By addressing malnutrition, infectious diseases and systemic implementation barriers, this framework seeks to create a healthier, more supportive school environment that enhances learner well-being and educational outcomes. Ultimately, the study contributes to regional efforts to strengthen school health systems in resource-limited settings across Africa.^11,13^

### Literature review

Effective school health policies are essential for promoting the well-being of learners and improving educational outcomes.^14^ Studies have emphasised that well-structured school health interventions can lead to better health outcomes and learning environments, yet Namibia’s current policies remain fragmented and lack a comprehensive framework.^13,15,16^

#### Malnutrition and infectious diseases

Malnutrition remains a significant issue in Namibia, adversely affecting cognitive development and educational achievement, with studies emphasising the importance of school feeding programmes and nutrition education.^1,3,17^ Likewise, infectious diseases such as HIV and acquired immunodeficiency syndrome (AIDS) and TB substantially contribute to absenteeism and hinder academic progress, highlighting the need for regular health screenings and preventive services.^9,10,11,18^

#### Global frameworks and local adaptation

Globally, frameworks such as the World Health Organization (WHO) Health-Promoting School Initiative offer comprehensive models.^13,18,19^ However, Namibia faces unique socioeconomic and geographical challenges that require adaptations of these international models.^13,20^ Research confirms that many schools in rural Namibia lack access to clean water, adequate sanitation and healthcare services, which diminishes the impact of generic interventions.^21,22^

#### Monitoring and evaluation and stakeholder engagement

Ongoing M&E is crucial for monitoring key health indicators such as malnutrition and absenteeism.^8,12^ Namibia currently lacks structured M&E systems, which hampers sustainability.^23^ Collaborative M&E involving educators, health professionals and communities is essential.^8^ In addition, engaging all stakeholders throughout policy development, implementation and evaluation ensures that school health policies are culturally appropriate and receive stronger community support.^8,9,12^

### Research question

*How can Namibia adapt global school health frameworks (WHO Health-Promoting Schools, Focusing Resources on Effective School Health [FRESH]) to ensure feasible implementation in resource-limited rural settings?*

### Aim

To develop and propose a comprehensive conceptual framework for strengthening school health policy in Namibia, focusing on evidence-based development, equitable implementation and robust M&E.

### Research objectives

- Identify policy gaps in Namibia’s current school health system and highlight areas needing improvement.
- Develop and assess a conceptual framework tailored to Namibia’s context for effective school health policy.
- Establish implementation and evaluation strategies to ensure sustainability and measurable outcomes of school health interventions.

## Research methods and design

This study employed a convergent mixed-methods design,^19^ integrating a systematic literature review with qualitative stakeholder consultations to develop a conceptual framework for strengthening school health policy in Namibia. The mixed-methods approach allowed for triangulation of evidence from published research and experiential insights from key stakeholders.

### Systematic review process

A systematic review was conducted following the Preferred Reporting Items for Systematic Reviews and Meta-Analyses (PRISMA) 2020 guidelines to identify evidence on school health policy development and implementation in Africa.^6,17,24,25^ The review protocol adhered to the PRISMA checklist and incorporated a flow diagram to illustrate the selection process.

#### Search strategy

**FIGURE 1:**
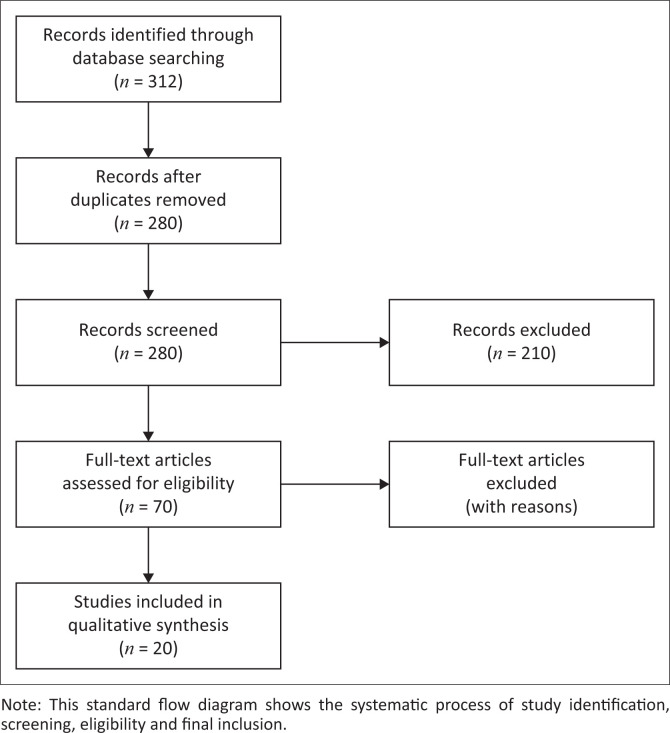
Preferred Reporting Items for Systematic Reviews and Meta-Analyses 2020 flow diagram for the systematic review process.

Electronic searches were performed in PubMed, Scopus, Google Scholar and World Health Organization Institutional Repository for Information Sharing (WHO IRIS) databases for articles published between 2010 and 2024 using keywords: ‘school health policy’, ‘health-promoting schools’, ‘Africa’, ‘education and health integration’, ‘Namibia’. Boolean operators (AND/OR) were applied to refine results (Figure 1 and Table 1).

**TABLE 1:** Included studies and eligibility notes.

Study identification	Author(s)	Year	Title	Source	Eligibility decision	Reason for inclusion
1	UNESCO	2025	Integrating health and well-being into education policy and planning: A handbook	UNESCO^16^	Included	Global policy relevance for school health integration
2	UNICEF Innocenti	2024	Improving education in Africa: Insights from research across 33 countries	UNICEF^7^	Included	Regional education-health linkage
3	Africa Centres for Disease Control and Prevention (CDC)	2025	Africa’s health security and sovereignty agenda: A new way forward	Africa CDC^8^	Included	Contextual health policy relevance
4	McLoughlin GM et al.	2022	Investigating implementation of school health policies through a health equity lens	Front Public Health^20^	Included	Focus on equity in school health policy
5	Khosla R, Brownb VT	2024	Implementation of sexual and reproductive health education policy in schools in Asia and Africa	International Journal of Sexual Health^17^	Included	Policy implementation in African schools
6	Adebayo A et al.	2023	School health and well-being in Nigeria: Gaps in policy and design	Journal of Public Health^1^	Included	African country case study on policy gaps
7	Department of Basic Education (DBE) & Health	2023	Implementation of the Integrated School Health Policy & Programme (ISHP)	South Africa Department of Basic Education & Health^26^	Included	Regional framework for school health
8	World Health Organization (WHO)	2023	Global standards for health-promoting schools	WHO^18^	Included	Benchmark for framework comparison
9	Global Partnership for Education (GPE)	2023	School health and nutrition: Policy guidance	GPE^21^	Included	Policy guidance for LMICs
10	World Bank	2023	Education and health integration in Sub-Saharan Africa	World Bank^13^	Included	Funding and sustainability insights
11	Mwangi S, Kamau M	2023	Strengthening school health programs in sub-Saharan Africa: Lessons from Kenya	Health Education Journal^6^	Included	Implementation lessons from Kenya
12	Kyere et al.	2020	Nutrition and cognitive development among African school-aged children	Nutrition Journal^2^	Included	Nutrition-health link in African schools
13	Degarege D et al.	2022	School health services in sub-Saharan Africa: Review of existing policies and challenges	BMC Public Health^11^	Included	Policy review and gaps
14	UNESCO Chair in Global Health and Education	2023	Transforming education through health and well-being	UNESCO^24^	Included	Integration of health in education globally
15	African Union (AU)	2023	Continental Education Strategy for Africa (CESA) and health integration	AU^3^	Included	Continental strategy alignment
16	Kruk et al.	2022	Education and health interlinkages in LMICs	Lancet^9^	Included	Evidence-based interlinkages
17	Dewey et al.	2025	Data-driven education policy for equity	Harvard University^14^	Included	Policy equity and a data-driven approach
18	UC Berkeley Center for African Studies	2024	Research on African education and health systems	UC Berkeley^23^	Included	Future research directions
19	WHO Africa	2024	School health and nutrition in Africa: Progress and gaps	WHO Africa^25^	Included	Regional progress and gaps
20	Global School Health Consortium	2023	Monitoring and evaluation frameworks for school health policies	Consortium^12^	Included	M&E framework relevance

Note: Please see the full reference list of the article, Katangolo-Nakashwa N, Mfidi FH, Mitonga KH. Strengthening school health in Namibia: A framework for policy development, implementation and evaluation. J Public Health Africa. 2026;17(1), a1584. https://doi.org/10.4102/jphia.v17i1.1584, for more information.

M&E, monitoring and evaluation; UNESCO, United Nations Educational, Scientific and Cultural Organization; UNICEF, United Nations Children’s Fund; LMIC, low- and middle-income country; BMC, BioMed Central; CEPR, Center for Education Policy Research; UC, University of California.

### Eligibility criteria

Inclusion: Peer-reviewed studies, reports and authoritative documents addressing school health policy frameworks, implementation strategies or M&E in African countries or LMICs.

Exclusion: Non-English publications, studies without policy relevance and those lacking full-text access.

#### Screening and selection

A total of 312 records were identified, duplicates removed (*n* = 32) and 280 titles and/or abstracts screened. Seventy full-text articles were assessed for eligibility, and 20 studies met the inclusion criteria. Reasons for exclusion included lack of policy focus or insufficient methodological detail.

#### Quality appraisal

Included studies were appraised using the Critical Appraisal Skills Programme (CASP) checklist for qualitative and mixed-methods research to ensure rigour and credibility.

#### Included studies

The final set of 20 studies comprised global and regional frameworks (e.g. WHO standards), African policy analyses and implementation case studies. These studies informed the conceptual framework development. A summary of included studies with eligibility notes is presented in Table 1.

### Qualitative component

Stakeholder perspectives were gathered through semi-structured interviews and focus group discussions (FGDs):

- Recruitment and sampling rationale: Participants (*n* = 30) were recruited through purposive sampling to ensure diversity of roles and experiences across the education and health sectors (educators, health professionals, policymakers and community representatives).
- Data saturation: Data saturation was achieved after 15 interviews and 3 FGDs (5 participants each), indicating no new policy-relevant information emerged.
- Trustworthiness: Thematic analysis was used. Trustworthiness was enhanced through intercoder reliability checks (consensus coding among two researchers), member validation (sharing emergent themes with participants for feedback) and researcher reflexivity (critical self-reflection on potential bias).^22^

#### Data analysis

**Qualitative data:** Thematic analysis was used to identify key themes, patterns and perceptions of Namibia school health policies from transcribed interviews and FGDs. Key themes included policy development gaps, resource allocation issues and school stakeholder engagement.

**Data integration:** Findings from the systematic review and qualitative analysis were integrated (triangulated) to develop a contextually relevant conceptual framework for school health policy in Namibia.

### Ethical considerations

Ethical clearance to conduct this study was obtained from the University of South Africa Research Ethics Committee (No. HSHDC/808/2017).

## Results

The findings of this study are presented as trends derived from the small stakeholder sample (*n* = 30). The development of the conceptual framework for strengthening school health policies in Namibia yielded significant findings, organised into the following key components: policy development, implementation strategies and M&E mechanisms. Each element was derived from the data gathered during the systematic literature review and stakeholder interviews.

### Policy development

A consistent trend indicated that existing school health policies in Namibia are fragmented and reactive rather than preventive.^25^

Current policies lack standardised guidelines for addressing malnutrition, mental health and infectious diseases in schools, according to stakeholders, particularly policymakers and educators (Table 1).

#### Evidence-based approach

A total of 85% of the interviewees stressed the importance of empirical evidence in school health policies. Existing policies often respond to crises such as disease outbreaks rather than preventing them.

#### Stakeholder inclusion

A total of 75% of focus group participants felt that communities, parents and learners were not sufficiently involved in policy development, resulting in policies that did not reflect local contexts and needs (Table 2).

**TABLE 2:** Components of the conceptual framework for school health policy in Namibia.

Component	Description
Policy development	Creating and implementing Namibia school health policies and guidelines based on evidence and comprehensive stakeholder input
Equitable resource allocation	Prioritising and distributing financial, infrastructural (WASH) and human resources to underserved, high-need rural schools
Human resource development	Enhancing educator and health professional capacity through specialised, ongoing training to implement school health policies effectively
School environment	Providing a healthy and supportive physical and social environment for students (e.g. sanitation, water access, psychological support)
Monitoring and evaluation	Implementing structured, collaborative systems to track key health indicators (e.g. absenteeism, malnutrition rates) and ensure continuous policy adjustment

WASH, water, sanitation and hygiene.

### Implementation strategies

Stakeholder discussions revealed resource constraints and capacity gaps as major barriers to implementation, especially in rural areas.

Many participants, especially school administrators and health professionals, cited resource and training shortages as policy implementation barriers (Figure 1).

#### Training and capacity building

**FIGURE 2:**
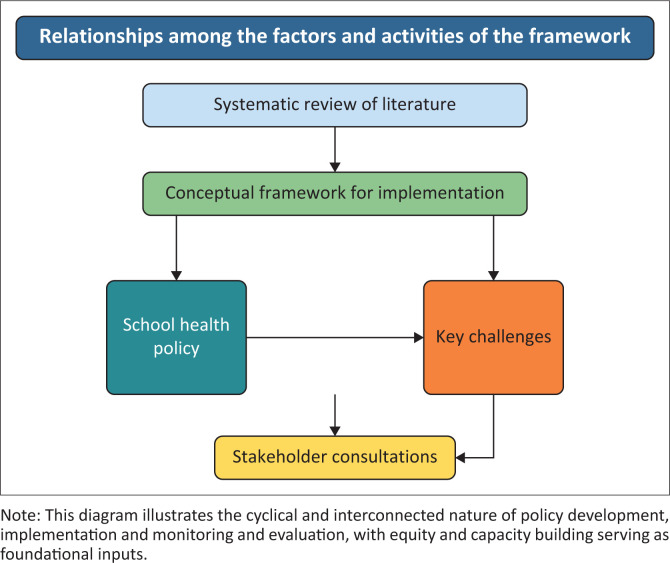
Relationships among the factors and activities of the framework.

Sixty-seven per cent of health professionals and educators reported inadequate school health intervention training. In rural areas, many schools lack physical and mental health staff. This caused inconsistent healthcare (Figure 2):

- Resource allocation: Urban and rural schools had very different resource distributions. Rural schoolchildren (78%) reported lacking clean water, toilets and regular medical screenings (Figure 2). Barriers: Figure 2: Barriers to school health policy implementation in Namibia visually highlights issues like insufficient funding and limited health services.

### Monitoring and evaluation mechanisms

The M&E mechanisms were found to be weak or absent in current policy frameworks.^23^

This study focused on (M&E) because school health policies are rarely rigorously evaluated (Figure 2). Participants said continuous assessment is needed to keep policies relevant and effective.

#### Outcome tracking

Only 45% of schools track health outcomes such as student absenteeism because of illness, malnutrition rates and school-based intervention effectiveness (Figure 2). Regular health monitoring improved attendance and performance in schools.

#### Collaborative monitoring and evaluation

Health agency stakeholders supported collaborative M&E involving educators, health professionals and community leaders (Figure 2).

### Conceptual framework

The final framework, detailed in Table 2, comprises three main components: Policy Development, Implementation Strategies, and M&E.

A conceptual model (Figure 1) shows how these three components interact to form a policy development, implementation and evaluation cycle. This model helps Namibian policymakers and educators improve school health. The model shows how to include appropriate M&E indicators in behaviour change.^15^

## Discussion

This conceptual framework for Namibia’s school health policy addresses critical gaps by emphasising evidence-based development, effective implementation and robust M&E.

### Addressing gaps in policy development

**FIGURE 3:**
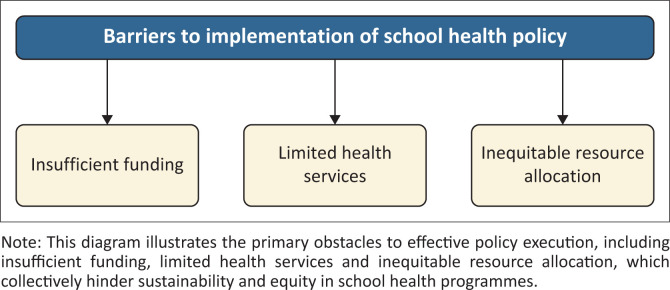
Barriers to school health policy implementation in Namibia.

The framework is explicitly designed to counter the fragmented and reactive nature of current policies. Its novelty lies in its contextual adaptation: it integrates the essential components of global frameworks (e.g. policy, environment, services) but enforces the use of stakeholder-informed data to prioritise resource distribution and capacity building (Figure 2 and Figure 3), which is critical for implementation success in countries like Namibia.^22,27^ This approach ensures that policy is not only conceptually sound but also practically feasible.

The proposed framework emphasises evidence-based approaches to ground policies in empirical data, which has been shown to improve policy effectiveness in other settings.^7,19,21^

Current policy development lacks a key component: parent, community and learner involvement.^28^ Health policy should involve stakeholders to create contextually relevant and sustainable interventions, according to research.^1,8,18^ The framework uses stakeholder feedback to ensure that policies reflect the local context, closing this gap (Table 1 and Figure 2).

### Challenges in implementation and feasibility

Implementation issues, particularly resource allocation and capacity building, dominated the study (Figure 3). The framework directly addresses the feasibility concern by suggesting prioritising underserved regions for resource allocation (e.g. clean water, sanitation) and creating specialised training programmes for educators, moving from an abstract policy to a tangible implementation strategy (Figure 3). This ensures that the framework is practical even in severely resource-constrained rural areas, focusing on incremental, high-impact improvements.

The framework suggests prioritising underserved regions for resource allocation to address these issues (Figure 3). This ensures the framework is practical even in severely resource-constrained rural areas, focusing on incremental, high-impact improvements.^12,22,27^

### Monitoring and evaluation

The framework’s emphasis on M&E is crucial, as many Namibia policies currently lack this process.^5^ Many Namibian school health policies lack a structured M&E process, resulting in poor implementation and little understanding of outcomes (Figure 1 and Table 1).^23^ Continuous monitoring of health indicators and intervention adjustments is stressed in the literature (Figure 2).^10^ This framework keeps health policies adaptable and effective by incorporating M&E. In Namibia, government agencies, schools and healthcare providers collaborate to implement health interventions, making this approach crucial (Figure 2).

The suggested *collaborative M&E approach* involving educators, health professionals and communities is supported by evidence of its success in improving accountability and sustainability in other LMICs.^1,8,18^

### Limitations and future research

The proposed framework addresses school health issues comprehensively, but its implementation is limited.

- Conceptual nature and generalisability: The framework is conceptual and has not been empirically tested in an intervention study. Furthermore, the qualitative data relies on self-reported stakeholder experiences, which may introduce bias. The small, purposively sampled size (*n* = 30) limits the generalisability of the derived trends to the entire country (Table 1).
- Future research: Future studies should focus on innovative, cost-effective health policy implementation in resource-constrained environments.^16,27^ Crucially, further research is needed to empirically assess this framework’s long-term effects on learner health and educational outcomes, ensuring its adaptability and efficacy in real-world settings (Figure 2).

### Recommendations

- Create evidence-based policies: Adapt school health policies to Namibia’s unique health challenges via empirical data.
- Equity in resource allocation: Increase funding for rural schools and enhancing access to basic health infrastructure, such as water and sanitation.
- Enhance capacity building: Train educators and health professionals to enhance school health interventions.
- Monitoring and evaluation: Robust M&E systems should be implemented to track and assess school health policies, enabling timely adjustments.
- Stakeholder engagement: Involve learners, parents, educators and communities in policy development and implementation for relevance and sustainability.

## Conclusion

This study provides a comprehensive conceptual framework for strengthening Namibia’s school health policies to address malnutrition, infectious diseases and mental health. The framework emphasises evidence-based policy development, equitable resource allocation, educator and health professional capacity building and robust M&E. By involving stakeholders in policymaking, the framework ensures health interventions are contextually relevant and sustainable. This framework has the potential to significantly improve learner well-being, reduce absenteeism and create a healthier, more supportive school environment in Namibia.
